# Fusion Gene KMT2A::SEPTIN6 in Acute Myeloid Leukemia Cell Line KOPM-88

**DOI:** 10.3390/cells15141286

**Published:** 2026-07-17

**Authors:** Stefan Nagel, Corinna Meyer, Maren Kaufmann, Silke Fähnrich, Ulfert Rand, Claudia Pommerenke, Roderick A. F. MacLeod, Sonja Eberth

**Affiliations:** 1 Department of Human and Animal Cell Lines, Leibniz-Institute DSMZ-German Collection of Microorganisms and Cell Cultures, 38124 Braunschweig, Germanysonja.eberth@dsmz.de (S.E.); 2 Department of Bioinformatics, Leibniz-Institute DSMZ-German Collection of Microorganisms and Cell Cultures, 38124 Braunschweig, Germany

**Keywords:** BMPR1B, EMX2, homeobox, homeodomain, MKX

## Abstract

**Highlights:**

**What are the main findings?**

- KOPM-88 contains the fusion gene KMT2A::SEPTIN6.
- KMT2A::SEPTIN6 activates BMP signalling and inhibits HOXA genes.

**What are the implications of the main findings?**

- KOPM-88 represents the first cell line model for this fusion gene.
- BMP signalling may serve as therapeutic target in KMT2A::SEPTIN6-positive acute myeloid leukemia (AML).

**Abstract:**

(1) Background: KMT2A (alias MLL) is located at chromosome 11q23 and encodes histone methyltransferase 2A which activates target genes via chromatin methylation of histone H3 lysine K4. Mutations of KMT2A, including partial tandem duplication (PTD) and fusion with partner genes, are present in both lymphoid and myeloid acute leukemia. More than 100 various KMT2A fusion genes have been described, with only a minority represented by cell line models. (2) Methods: Cytogenetic and genomic copy number analyses, PCR, Western blot and RNA-sequencing were performed to characterize aberrations in acute myeloid leukemia (AML) cell line KOPM-88. Bioinformatic analysis of public AML patient data revealed differentially expressed genes. Functional analyses were performed by siRNA-mediated knockdown and live-cell imaging. (3) Results: The AML cell line KOPM-88 is derived from a boy at relapse and has been reported to carry t(X;11)(q24;q23), albeit with uncharacterized breakpoints. In KOPM-88 we identified fusion gene KMT2A::SEPTIN6 generated by this translocation, but excluded KMT2A-PTD. KMT2A::SEPTIN6 activated bone morphogenetic protein (BMP)-signalling and inhibited expression of HOXA7 and HOXA9. BMP signalling in turn activated cell proliferation and inhibited CDKN2B expression. (4) Conclusions: KOPM-88 contains fusion gene KMT2A::SEPTIN6, representing the only cell line model for this rare type of KMT2A rearrangement. KOPM-88 may serve to advance novel therapeutic treatments for KMT2A::SEPTIN6-positive AML.

## 1. Introduction

KMT2A, formerly known as MLL, encodes histone methyltransferase 2A, which adds up to three methyl groups to lysine K4 of histone H3. This modification encourages chromatin opening and gene activation [1]. KMT2A operates in a massive complex, containing several proteins, including AFF1 and MENIN. Functionally, KMT2A is the counterpart of polycomb repressor complexes, which inhibit gene expression via methylation of other histone lysines [1]. These different histone modifications have been collated to delineate a histone code, which covers concomitant transcriptional gene regulation in all cell types including hematopoietic cells and tissues [2].

The original name MLL stands for mixed lineage leukemia to reflect the presence of mutations in both lymphoid and myeloid leukemia [1]. The gene KMT2A is located at chromosomal position 11q23 and is frequently involved in translocations, generating fusion genes. To date, 107 different fusion partner genes have been described, thus representing the conspicuous heterogeneity of KMT2A-derived oncogenes [3]. However, classification of these partner genes revealed just five groups, which markedly differ in their incidence. Group 1 contains the most frequent fusion partners, those that encode nuclear factors associated with KMT2A in a complex, including AFF1/AF4, MLLT3/AF9 and MLLT1/ENL. Genes of Group 2 encode proteins containing a coiled-coil domain. Group 3 contains SEPTIN genes and Group 4 histone acetyltransferases, while KMT2A containing an internal partial tandem duplication (PTD) constitutes Group 5 [1].

KMT2A fusion genes operate as oncogenes in acute leukemia. Target gene analyses revealed aberrant activation of hematopoietic differentiation genes including MEIS1 and members of the HOXA gene cluster [4,5]. HOXA homeobox genes are physiological target genes of KMT2A in hematopoiesis and correspondingly deregulated by mutated KMT2A [2]. Treatments of patients with KMT2A fusions usually target complex members or recruited co-factors like MENIN or DOT1L, avoiding the problem of fusion gene heterogeneity [6]. Despite existing therapeutic strategies for patients with KMT2A rearrangements, their diversity and corresponding prognostic differences necessitate intensified research into the underlying oncogenic mechanisms.

Leukemia/lymphoma cell lines often contain key recurrent chromosomal aberrations, confirming their fitness as models to investigate their pathologic function and treatment options [7,8]. Cell lines containing KMT2A rearrangements mainly cover the most frequent Group 1, while none are available for Group 3, containing fusions between KMT2A and selected SEPTIN genes [9]. Here, we characterized AML cell line KOPM-88 and identified the fusion gene KMT2A::SEPTIN6 therein. Functional analyses indicated a role in activation of the bone morphogenetic protein (BMP) pathway and cell proliferation, while excluding HOXA gene activation. Thus, KOPM-88 is the first cell line model shown to contain this hitherto unrepresented type of KMT2A rearrangement and may help to illuminate its pathology and to develop therapeutic treatments in vitro.

## 2. Materials and Methods

### 2.1. Cell Lines and Treatments

AML cell lines EOL-1 (ACC 386), HL-60 (ACC 3), KASUMI-1 (ACC 220), KG-1 (ACC 14), KOPM-88 (ACC 11029), MONO-MAC-6 (ACC 124), NB-4 (ACC 207), OCI-AML3 (ACC 582) and THP-1 (ACC 16) were used in this study (German Collection of Microorganisms and Cell Cultures DSMZ, Braunschweig, Germany). They are cultivated as described on the website (www.dsmz.de). Of note, KOPM-88 was cultivated in RPMI1640 supplemented with 10% FBS and 10% 5637-supernatant. All cell lines had been authenticated and tested negative for mycoplasma infection by our in-house service.

KOPM-88 cells were stimulated with 20 ng/mL BMP2 and BMP4 (R&D Systems, Wiesbaden, Germany). Modification of selected gene expression levels was performed using gene-specific siRNA oligonucleotides with reference to AllStars negative Control siRNA (siCTR) obtained from Qiagen (Hilden, Germany). SiRNAs (80 pmol) were transfected into 1 × 10^6^ cells by electroporation using the EPI-2500 impulse generator (Fischer, Heidelberg, Germany) at 350 V for 10 ms. Electroporated and stimulated cells were harvested after 20 h cultivation.

Functional analyses of KOPM-88 cells treated for gene knockdown were performed using the IncuCyte S3 Live-Cell Imaging Analysis System (Sartorius, Göttingen, Germany). Cells were additionally treated with 100 µM etoposide dissolved in DMSO, obtained from Sigma-Aldrich (Taufkirchen, Germany). Apoptotic cells were detected using the IncuCyte Caspase-3/7 Green Apoptosis Assay diluted at 1:2000 (Sartorius). Data analysis was performed using the Cell-by-Cell software tool (Sartorius, Incucyte 2022B Rev2). Live-cell imaging experiments were performed twice with fourfold parallel tests.

### 2.2. Polymerase Chain-Reaction (PCR) Analyses

Total RNA was extracted from cultivated cell lines using TRIzol reagent (Invitrogen, Darmstadt, Germany) and 1 µg was applied for cDNA synthesis, using random priming and Superscript II (Invitrogen).

Fusion transcripts were detected by reverse transcription (RT)-PCR, using oligonucleotides obtained from Eurofins (Ebersberg, Germany): ETV6-F 5′-AGGCCAATTGACAGCAACAC-3′ (located in exon 3), ETV6-R 5′-TGCACATTATCCACGGATGG-3′ (located in exon 5), KMT2A-F2 5′-CCACTCCTAGTGAGCCCAAG-3′ (located in exon 7), KMT2A-R2 5′-CTGAAGGAGACCTTGTGGGAC-3′ (located in exon 2), SEPTIN6-F 5′-GTTTCCTGTGCAGTAGCTCCCG-3′ (located in exon 1), and SEPTIN6-R 5′-GCACAGGATGTTGAAGCAGAAGCC-3′ (located in exon 2). PCR was performed using taqpol (Qiagen) and thermocycler TGradient (Biometra, Göttingen, Germany). PCR products were analyzed by gel electrophoresis and documented with the Azure c200 Gel Imaging System (Azure Biosystems, Dublin, CA, USA), cloned into vector pCR4-TOPO (Invitrogen) and sequenced at Eurofins.

Real-time quantitative (RQ)-PCR analyses were performed using the 7500 Real-time System and the ddCt-method, commercial buffer and taqman primer sets (Applied Biosystems/Life Technologies, Darmstadt, Germany), or custom designed oligonucleotides as described above. Normalization of expression levels was performed by quantification of the TATA box binding protein (TBP) transcripts. Experimental analyses were performed as biological triplicates; RQ-PCR analyses were performed in triplicate. Standard deviations are indicated in the figures as error bars, showing a representative result. Statistical significance was assessed by the *t*-test. The calculated *p*-values are indicated by asterisks (* *p* < 0.05, ** *p* < 0.01, *** *p* < 0.001, n.s. = not significant).

### 2.3. Protein Analysis

For the analysis of selected surface markers, cells were stained with primary antibodies from BD Biosciences (Heidelberg, Germany), detecting CD3 (clone SK7), CD4 (clone SK3), CD13 (clone Leu-M7), CD15 (clone MMA), CD19 (clone 4G7), CD33 (clone HIM3-4), CD34 (clone 581), and HLA-DR (clone Tu39), and from Beckman Coulter (Krefeld, Germany), detecting CD14 (clone RMO52). After washing, cells were stained with FITC-labelled secondary antibody goat anti-mouse IgG/IgM (BD Biosciences). Dead cells were excluded by propidium iodide, and staining and measurements were conducted using CytoFLEX S (Beckman Coulter). Data analysis was performed with FlowJo v10 (BD Biosciences). Flow cytometry analysis of KOPM-88 confirmed the presence of surface markers CD4, CD13 and CD33, and the absence of CD3, CD14, CD19, CD34 and HLA-DR [10]. Slight positivity was detected for CD14 and CD15 (Figure S1).

We used the SIGMAFast protease inhibitor cocktail (Sigma-Aldrich) to prepare protein lysates from cell lines for subsequent Western blot analysis. Proteins were transferred onto nitrocellulose membranes (Bio-Rad, Munich, Germany) using the semi-dry method. The blots were blocked with 5% dry milk powder which was dissolved in phosphate-buffered-saline buffer (PBS). The following antibodies were used: alpha-Tubulin (Sigma, #T6199), ERK (Santa Cruz Biotechnology, Heidelberg, Germany, #sc-393713), SEPTIN6 (Santa Cruz Biotechnology, #sc-271459), and KMT2A (Santa Cruz Biotechnology, #sc-271459). For loading control blots were first reversibly stained with Poinceau (Sigma-Aldrich). Detection of alpha-Tubulin (TUBA) was performed thereafter. Secondary antibodies (linked to peroxidase) were used for detection by Western Lightning ECL (Perkin Elmer, Waltham, MA, USA). We used the digital system ChemoStar Imager (INTAS, Göttingen, Germany) to document the data.

### 2.4. Cytogenetic and Genomic Profiling Analysis

Karyotyping and fluorescence in situ hybridization (FISH) were performed as described previously [11]. Whole chromosome painting probes were obtained from KaryoGenix (Edingen-Neckarhausen, Germany). Fluorescent images were captured with an Axion A1 microscope using the Axiocam 208 Color and ZEN 3.3 blue edition software (Zeiss, Göttingen, Germany).

For genomic profiling, genomic DNA of the cell lines KOPM-88 and EOL-1 was prepared using the QIAamp DNA mini-kit (Qiagen). Labelling, hybridization and scanning of Cytoscan HD arrays were performed by ATLAS Biolabs (Berlin, Germany). We used the Chromosome Analysis Suite software version 3.1.0.15 (Affymetrix, High Wycombe, UK) to interpret the data and to determine copy number alterations accordingly.

### 2.5. RNA-Sequencing and Bioinformatic Analyses

Total RNA from KOPM-88 was extracted using the miRNeasy mini-kit (Qiagen) including Dnase digestion. RNA quality was measured using the Agilent 2100 Bioanalyzer (Agilent Technologies, Waldbronn, Germany), revealing RIN values of 9.8. Sample libraries were prepared and sequenced by NOVOGENE (Munich, Germany) with the NGS Stranded RNA Library Prep Set (PT044) by aiming for a minimum of 30 M 2 × 150 bp read pairs per sample with an insert size of >150 bp in order to reduce overlapping paired-end read sequences [12]. The library was checked by Qubit and real-time PCR for quantification and Agilent Fragment Analyzer for size distribution detection. Quantified libraries were pooled and sequenced on Illumina NovaSeq X Plus, San Diego, CA, USA, according to effective library concentration and data amount. The search for potential fusion genes was performed with fusion-catcher [13]. Data were deposited at ArrayExpress (www.ebi.ac.uk/biostudies/arrayexpress, accessed on 20 May 2026) and are available via E-MTAB-17091.

We analyzed 42 pediatric AML patients with KMT2A rearrangements, using the public GEO dataset GSE19577 [14] and the associated online tool GEO2R [15].

## 3. Results

### 3.1. Identification of Fusion Gene KMT2A::SEPT6 in KOPM-88

**Figure 1:**
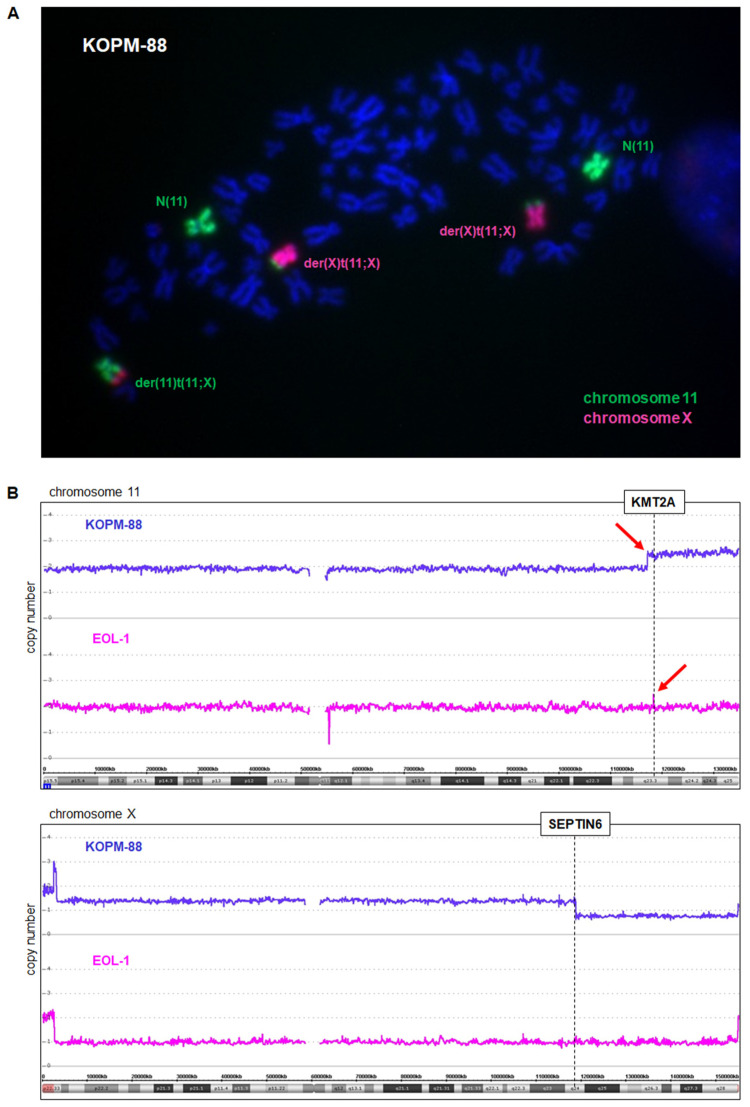
Cytogenetic and genomic profiling analyses of KOPM-88. (**A**) FISH analysis of metaphase chromosomes of KOPM-88 using whole chromosome painting probes for chromosome 11 (green) and chromosome X (pink). (**B**) Copy number variations obtained by genomic profiling analysis of KOPM-88 and EOL-1 showing data for chromosome 11 (above) and chromosome X (below). The gene loci for KMT2A and SEPTIN6 are indicated. Red arrows highlight a centromeric breakpoint in KOPM-88 reflecting the need for genomic inversion to correctly frame the gene fusion unlike KMT2A, and the KMT2A-PTD in EOL-1 absent in KOPM-88.

KOPM-88 is an AML cell line established by Hayashi and coworkers (2007) from the peripheral blood of an eleven-year-old boy at relapse. Reported cytogenetic analysis of KOPM-88 showed a near-tetraploid karyotype with additional alterations, including loss of chromosome 1p and chromosomal aberration t(X;11)(q24;q23). Furthermore, the authors described the presence of KMT2A-PTD, highlighting KMT2A mutation in this cell line [10]. Here, we were interested in identifying the genes targeted by t(X;11)(q24;q23). Cytogenetic and FISH analyses confirmed the tetraploid karyotype and the presence of t(X;11) (Figure 1A). Using whole chromosome painting probes, our data showed two normal chromosomes 11, two aberrant chromosomes X, each containing material from chromosome 11, and one chromosome 11 containing material from chromosome X. Copy number quantification via SNP-array analysis confirmed loss of chromosome 1p and demonstrated additional genomic aberrations (Figures S2 and S3A). Furthermore, these data indicated a combination of chromosomal breakpoints with copy number alterations at 11q23 located between the genes RNF214 and CEP164, and at Xq24 located within intron 1 of SEPTIN6 Figure S3B).

**Figure 2:**
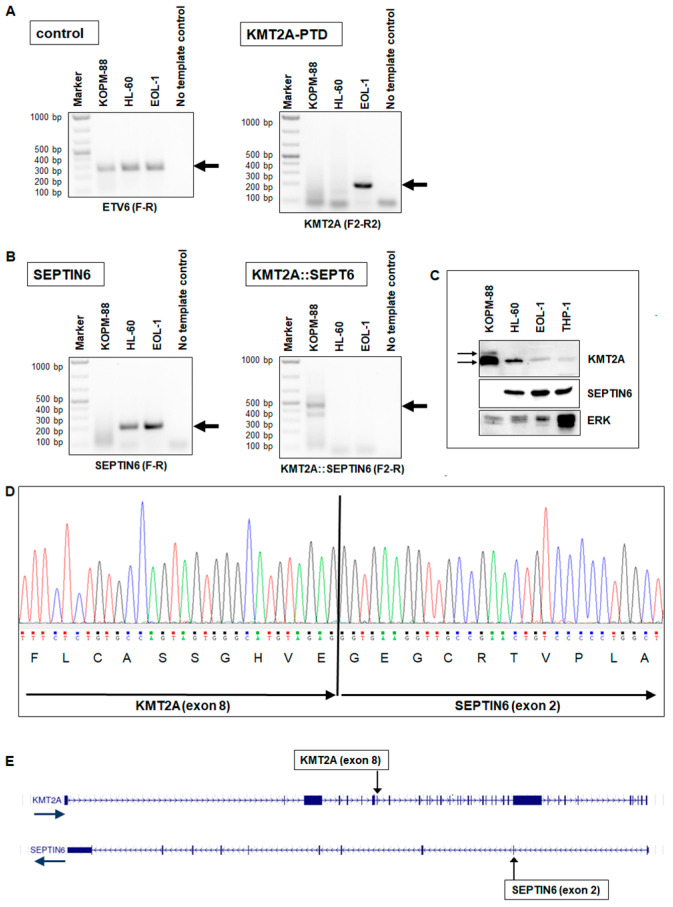
Identification of fusion gene KMT2A::SEPTIN6 in KOPM-88. RT-PCR analysis of AML cell lines KOPM-88, HL-60 and EOL-1 for (**A**) ETV6, serving as control, and KMT2A-PTD, and (**B**) SEPTIN6 and fusion transcript KMT2A::SEPTIN6. Corresponding PCR products are indicated by arrows. (**C**) Western blot analysis of AML cell lines KOPM-88, HL-60, EOL-1 and THP-1 for KMT2A, SEPTIN6 and ERK, serving as control. (**D**) Sequencing results for PCR product KMT2A::SEPTIN6 from KOPM-88, identifying fusion between exon 8 of KMT2A and exon 2 of SEPTIN6 in KOPM-88. (**E**) Schematic presentation of the genes KMT2A (above) and SEPTIN6 (below) obtained from the UCSC genome browser, highlighting the fused exons and demonstrating their opposing genomic orientation.

RT-PCR analysis of KOPM-88 in comparison to the AML cell lines HL-60 and EOL-1 confirmed the reported KMT2A-PTD in EOL-1 [16], but showed its exclusion in KOPM-88 (Figure 2A). Of note, the KMT2A primers used bind within exons 2 and 7, suitable for detecting alternative KMT2A-PTD variants as well. Copy number analysis confirmed these results for EOL-1 and KOPM-88 (Figure 1B). However, a potential rearrangement of KMT2A and the identified copy number alteration within intron 1 of SEPTIN6 prompted the search for fusion gene KMT2A::SEPTIN6 in KOPM-88, which has been reported in pediatric AML patients, fusing KMT2A exon 7, 8 or 9 with SEPTIN6 exon 2 [17,18,19,20,21,22]. RT-PCR analysis demonstrated loss of normal SEPTIN6 expression and the presence of a potential fusion transcript in KOPM-88 (Figure 2B). Western blot analysis confirmed the absence of the SEPTIN6 protein and an additional band above normal KMT2A in KOPM-88 (Figure 2C). Sequence analysis of the prominent PCR fusion product after cloning and Sanger sequencing demonstrated the presence of fusion transcript KMT2A::SEPTIN6, while the faint PCR product below emerged as an artefact. The fusion sequence revealed a combination of exon 8 from KMT2A to exon 2 from SEPTIN6 (Figure 2D). Moreover, RNA-sequencing data for KOPM-88 confirmed the presence of fusion transcripts KMT2A::SEPTIN6 and failed to detect KMT2A-PTD transcripts. Thus, we identified the fusion gene KMT2A::SEPTIN6 in AML cell line KOPM-88 while excluding the reported KMT2A-PTD.

FISH analysis, RT-PCR and RNA-sequencing demonstrated that chromosomal aberration t(X;11)(q24;q23) generated the fusion gene KMT2A::SEPTIN6. Our copy number data showed an additional chromosomal breakpoint at 11q23, located centromeric of KMT2A (Figure 1B and Figure S3B). Together, these results indicated a more complex rearrangement at 11q23, adopting the configuration necessary to generate a correctly orientated fusion gene (Figure 2E).

### 3.2. Functional Analyses of KMT2A::SEPT6 in AML

**Figure 3:**
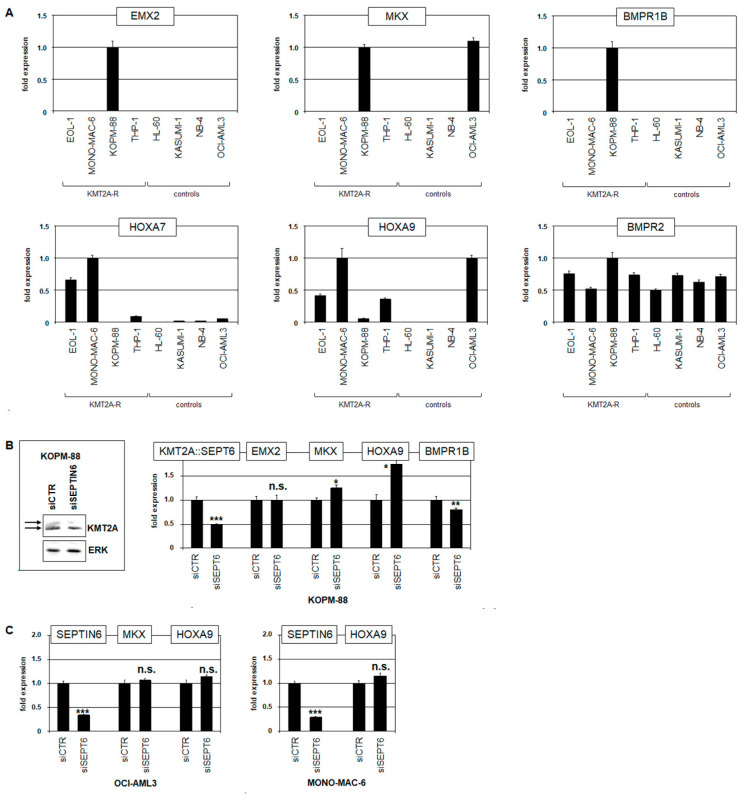
Target gene analysis of KMT2A::SEPTIN6. (**A**) RQ-PCR analyses of selected AML cell lines for EMX2, MKX, HOXA7, HOXA9, BMPR1B and BMPR2, showing their fold expression according to the cell line with the highest level. (**B**) Western blot analysis of KMT2A and ERK, serving as control, in KOPM-88 treated for knockdown of SEPTIN6/KMT2A::SEPTIN6 (left). RQ-PCR analyses of EMX2, MKX, HOXA9 and BMPR1B in KOPM-88 cells treated for KMT2A::SEPTIN6 knockdown. (**C**) RQ-PCR analyses of MKX and HOXA9 in OCI-AML3 cells (left) and of HOXA9 in MONO-MAC-6 cells treated for SEPTIN6 knockdown. Statistical significance was assessed by *t*-test, and the calculated *p*-values are indicated by asterisks (* *p* < 0.05; ** *p* < 0.01; *** *p* < 0.001; n.s. not significant).

To reveal potential target genes of fusion protein KMT2A::SEPTIN6 we analyzed public expression profiling dataset GSE19577, covering 42 pediatric AML patients with KMT2A rearrangements including three patients with KMT2A::SEPTIN6. Using the associated online tool GEO2R we compared these three patients with the other patients harbouring different KMT2A rearrangements (Figure S4, Table S1). The data showed that in KMT2A::SEPTIN6-positive patients SEPTIN6 was downregulated, while homeobox genes EMX2 and MKX in addition to BMP-receptor BMPR1B were upregulated. In addition, analysis of HOXA7 in this dataset showed reduced expression in KMT2A::SEPTIN6-positive patients (Figure S4). To relate these clinical data with cell lines we quantified the expression of these genes in AML cell lines carrying KMT2A rearrangements, including EOL-1 (KMT2A-PTD), MONO-MAC-6 (KMT2A::MLLT3), KOPM-88 (KMT2A::SEPTIN6) and THP-1 (KMT2A::MLLT3), and four controls without KMT2A rearrangements (HL-60, KASUMI-1, NB-4 and OCI-AML3). The results demonstrated elevated expression of EMX2, MKX and BMPR1B for KOPM-88, while HOXA7 was reduced. Furthermore, we showed enhanced BMPR2 and downregulated HOXA9 (Figure 3A). Thus, in both KMT2A::SEPTIN6-positive AML patients and cell line KOPM-88, BMP-receptors and homeobox genes EMX2 and MKX were activated, while homeobox gene HOXA7 was downregulated.

To examine the potential regulatory impact of fusion gene KMT2A::SEPTIN6 on selected gene candidates, we performed siRNA-mediated knockdown experiments in KOPM-88, which has lost its normal SEPTIN6 genes (Figure 2B). Therefore, siRNA-mediated targeting of SEPTIN6 exclusively resulted in the downregulation of fusion gene KMT2A::SEPTIN6. This treatment effected a slight increase in MKX expression, and enhanced HOXA9 expression and downregulation of BMPR1B, while EMX2 was not affected. Thus, fusion gene KMT2A::SEPTIN6 activated BMP-receptor BMPR1B and inhibited homeobox genes HOXA9 and MKX.

To analyze the regulatory role of SEPTIN6 in AML we performed its siRNA-mediated knockdown in cell line OCI-AML3 which harbours normal KMT2A and SEPTIN6 genes and expresses homeobox genes MKX and HOXA9. Quantification of both KMT2A::SEPTIN6 target genes showed that SEPTIN6 neither regulated MKX nor HOXA9 (Figure 3C). Furthermore, knockdown of SEPTIN6 in MONO-MAC-6 harbouring KMT2A::MLLT3 also showed absent regulation of HOXA9 (Figure 3C). Thus, deregulation of MKX and HOXA9 in AML is independent of SEPTIN6.

**Figure 4:**
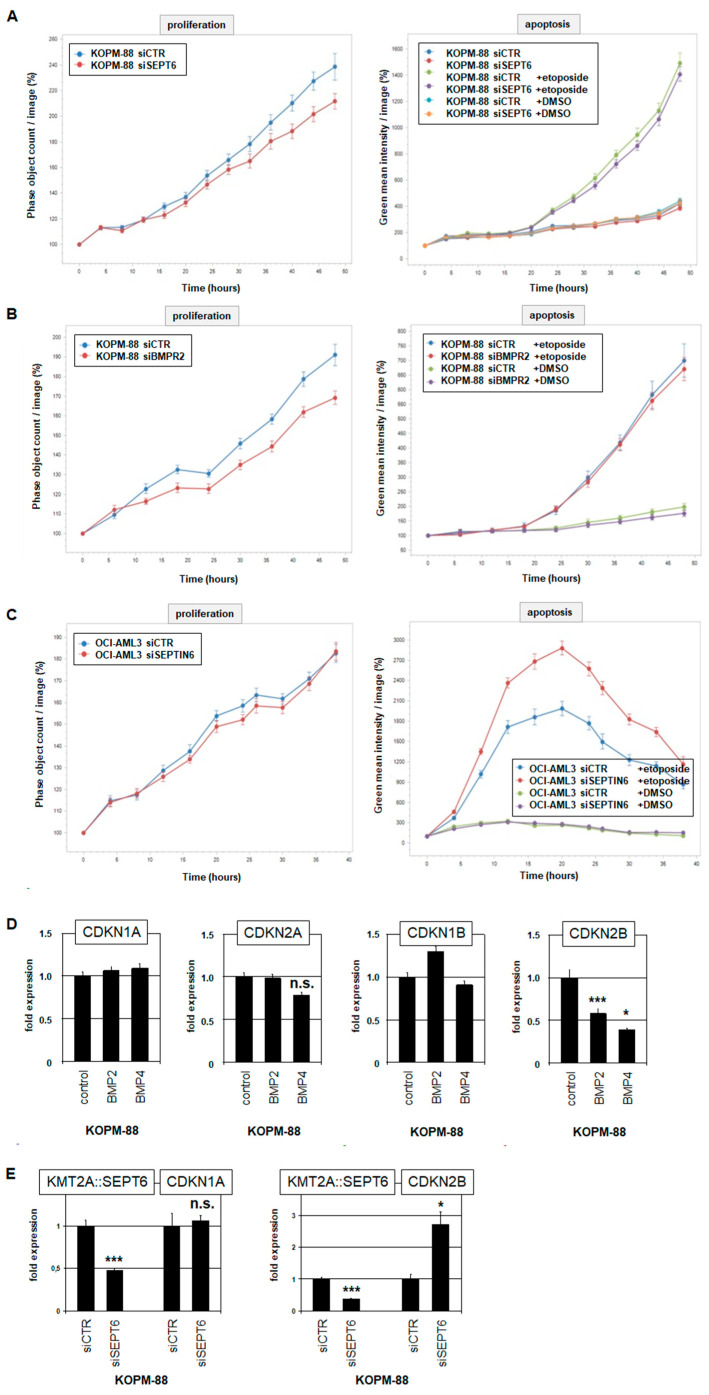
Functional analyses of KMT2A::SEPTIN6 in KOPM-88. Live-cell imaging analyses of KOPM-88 cells treated for siRNA-mediated knockdown of (**A**) SEPTIN6 and (**B**) BMPR1B, and with etoposide. (**C**) Live-cell imaging analyses of OCI-AML3 cells treated for siRNA-mediated knockdown of SEPTIN6 and with etoposide. (**D**) RQ-PCR of KOPM-88 stimulated with BMP2 and BMP4 for CDKN1A, CDKN2A, CDKN1B and CDKN2B. (**E**) RQ-PCR analysis of CDKN1A (left) and CDKN2B (right) in KOPM-88 treated for siRNA-mediated knockdown of SEPTIN6/KMT2A::SEPTIN6. Statistical significance was assessed by *t*-test, and the calculated *p*-values are indicated by asterisks (* *p* < 0.05; *** *p* < 0.001; n.s. not significant).

Functional analysis of KMT2A::SEPTIN6 was performed by live-cell imaging of KOPM-88 cells treated for SEPTIN6 knockdown. The data indicated that KMT2A::SEPTIN6 may support cell proliferation without regulating apoptosis as analyzed by the addition of apoptosis-inducer etoposide (Figure 4A). Interestingly, siRNA-mediated knockdown of the general BMP-receptor BMPR2 showed similar results for proliferation and apoptosis (Figure 4B), indicating that KMT2A::SEPTIN6 may support proliferation via activation of BMP signalling. In contrast, live-cell imaging of OCI-AML3 cells treated for knockdown of SEPTIN6 indicated that SEPTIN6 supports survival but has no impact on proliferation (Figure 4C). Collectively, these data may show that the generation of fusion gene KMT2A::SEPTIN6 and the loss of SEPTIN6 differ functionally in AML.

Cyclin-dependent kinase inhibitors (CDKNs) are key regulators of cell proliferation and their deregulation has been described in the context of KMT2A rearrangements [23,24,25]. According to these reported data, stimulation of KOPM-88 with BMP2 or BMP4 reduced expression of cell cycle inhibitor CDKN2B, while sparing CDKN1A, CDKN1B and CDKN2A (Figure 4C). Moreover, knockdown of KMT2A::SEPTIN6 raised expression of CDKN2B while sparing CDKN1A (Figure 4D). Taken together, these functional analyses showed that KMT2A::SEPTIN6 activated BMP signalling which in turn reduced expression of cell cycle inhibitor CDKN2B. This regulatory connection may underlie the observed KMT2A::SEPTIN6-mediated support of cell proliferation.

## 4. Discussion

**Figure 5:**
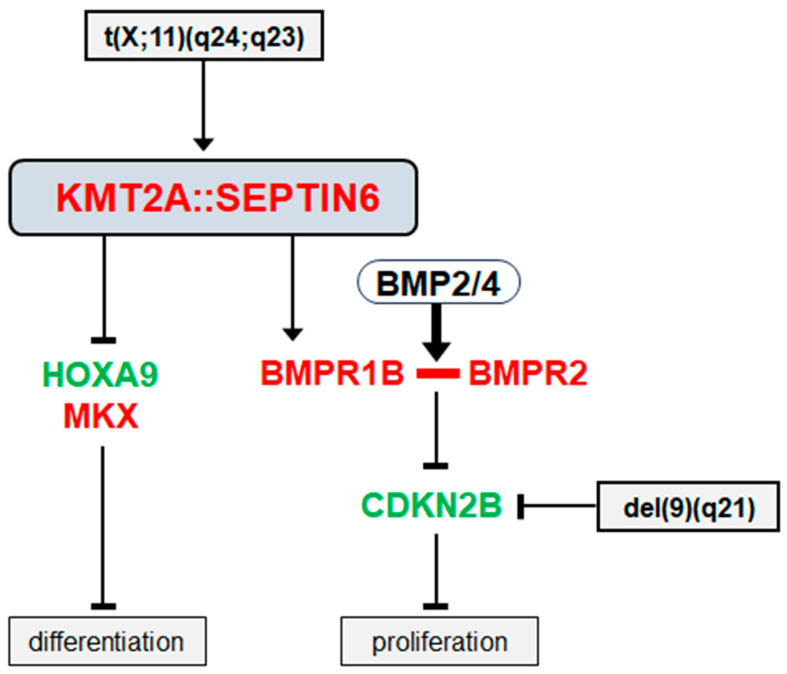
Study summary. This diagram summarizes the results of this study generated in AML cell line KOPM-88. The colour of the genes indicates their expression level in the cell line: red-labelled genes are highly expressed, green genes are downregulated.

In this study, cytogenetic, genomic, and molecular characterization of AML cell line KOPM-88 identified fusion gene KMT2A::SEPTIN6 therein. Functional analyses revealed activation of BMP signalling and inhibition of homeobox genes HOXA7, HOXA9 and MKX. Figure 5 summarizes these results which are discussed below.

KMT2A rearrangements are frequent and of diagnostic importance in both myeloid and lymphoid acute leukemia [3,26]. They fall into five groups according to the type of fusion partner and the presence of an internal partial duplication of KMT2A itself [1], each differing in incidence and prognosis. Therefore, clinical identification and molecular investigation of their pathological activities are key to improve the treatment of affected patients.

In contrast to the report of Hayashi [10], we excluded the presence of KMT2A-PTD in KOPM-88 deposited at the DSMZ, while instead confirming reported expression of surface markers and the presence of specific cytogenetic abnormalities, which together confirm cell line authenticity. Most importantly, we identified fusion gene KMT2A::SEPTIN6 which is formed by the reported aberration t(X;11)(q24;q23). Therefore, we conclude that cell line KOPM-88 contains fusion gene KMT2A::SEPTIN6 as the sole KMT2A rearrangement. The fusion gene KMT2A::SEPTIN6 has been exclusively described in pediatric AML patients, consistent with the reported origin of KOPM-88 that has been established from an eleven-year-old boy with AML [10,27].

Particular KMT2A rearrangements including KMT2A::MLLT3 mediate inhibition of the myeloid differentiation programme [28]. Accordingly, reported target genes are differentiation factors including activated HOXA genes and MEIS1 [4,5,16]. In contrast, our data showed that KMT2A::SEPTIN6 inhibited the homeobox genes HOXA7 and slightly inhibited MKX in AML patients and cell line KOPM-88 alike. Nevertheless, MKX is highly expressed in patients and KOPM-88, representing a TALE-class homeobox gene which has recently been described as an oncogene in AML [29]. Thus, aberrant MKX expression may play a pathogenic role in KMT2A::SEPTIN6-positive AML, but its heightened activity is actually decreased by formation of this fusion gene. EMX2 is a member of the NKL-class of homeobox genes and no target of KMT2A::SEPTIN6, but aberrantly expressed in subsets of AML patients with normal karyotype [30]. Collectively, expression and deregulation of homeobox genes is different in KMT2A::SEPTIN6-positive cases as compared to group 1 cases. This difference may underlie the reportedly good prognosis of pediatric AML patients expressing fusion gene KMT2A::SEPTIN6 [31].

The human genome contains 14 SEPTIN genes which encode GTP-binding proteins that associate with membranes and filaments of the cytoskeleton [27]. Five SEPTIN family genes reportedly undergo fusion to KMT2A in AML, including SEPTIN2, SEPTIN5, SEPTIN6, SEPTIN9 and SEPTIN11 [27]. These fusion genes form the Group 3 of KMT2A partner genes and are quite rare [1]. Fused SEPTINS retain their coiled-coil domain, which may support dimerization and activation of the corresponding fusion proteins [32]. Furthermore, SEPTIN-fusions are frequently associated with downregulation of the corresponding SEPTIN genes [27]. This relationship was confirmed in the KMT2A::SEPTIN6-positive AML patients and in the cell line KOPM-88 analyzed here, which may thus legitimately be used to investigate the role of SEPTIN6 in AML.

Our data indicated that KMT2A::SEPTIN6 activates the BMP signalling pathway via BMP-receptor BMPR1B. Furthermore, we showed that BMP signalling inhibited expression of cell cycle inhibitor CDKN2B. BMPR1B is a type 1 receptor that forms a heteroduplex with type 2 receptor BMPR2. Upon BMP-binding, these receptors activate SMAD transcription factors which translocate into the nucleus and directly regulate target genes [33]. SMAD4 reportedly inhibits CDKN2B in endothelial cells [34], supporting our findings of BMP-mediated CDKN2B regulation. Repressed expression of cell cycle inhibitors is associated with KMT2A fusion genes, which directly or indirectly regulate their target genes [23,24,25]. Deregulation of the BMP pathway plays an oncogenic role in several types of cancer including AML. The availability of BMP pathway inhibitors may, thus, provide a promising treatment option for KMT2A::SEPTIN6-positive AML patients [35]. However, fusion gene KMT2A::SEPTIN6 may additionally deregulate other genes and pathways which remain to be identified and may offer alternative therapy options.

The generation of fusion gene KMT2A::SEPTIN6 results in loss of SEPTIN6. Our analysis of SEPTIN6 downregulation in AML cell line OCI-AML3 showed the absence of homeobox gene deregulation but increased apoptosis. Thus, fusion gene KMT2A::SEPTIN6 and SEPTIN6 downregulation showed no functional similarities, highlighting the oncogenic role of KMT2A::SEPTIN6.

Leukemia/lymphoma cell lines serve as diagnostic tools and as models to investigate pathologic mechanisms and their therapeutic targeting [7]. Valuable examples for cell lines with fusion genes are K-562 (containing BCR::ABL1), KASUMI-1 (RUNX1::RUNX1T1), NB-4 (PML::RARA), and THP-1 (KMT2A::MLLT3) [8]. Drexler listed 39 cell lines containing KMT2A rearrangements, including 17 lymphoid and 22 myeloid cell lines [9]. However, most of their fusion genes belong to Group 1 of KMT2A rearrangements, while no Group 3 cell line has been reported so far. Thus, KOPM-88 is the only cell line model for fusion gene KMT2A::SEPTIN6 currently available and may, therefore, be useful to advance basic research and pre-clinical studies in this poor-prognosis entity, characterized by chemotherapy resistance and high relapse rates.

## 5. Conclusions

We identified fusion gene KMT2A::SEPTIN6 in AML cell line KOPM-88. KOPM-88 is first cell line model for this leukemic entity and should prove a useful asset for investigating its oncogenic function and seeking novel treatment options.

## Data Availability

Data were deposited at ArrayExpress (www.ebi.ac.uk/biostudies/arrayexpress, accessed on 20 May 2026) and are available via E-MTAB-17091.

## Supplementary Materials

The following supporting information can be downloaded at: https://www.mdpi.com/article/10.3390/cells15141286/s1, Figure S1: Flow cytometry analysis of KOPM-88 for CD3, CD4, CD13, CD14, CD15, CD19, CD33, CD34 and HLA-DR; Figure S2: Genomic profiling data for KOPM-88, showing the copy number states of all chromosomes; Figure S3: Genomic profiling data for KOPM-88, showing the copy number states of (A) chromosome 1 (above) and chromosome 9 (below), and (B) of enlarged parts of chromosome 11 (above) and chromosome X (below). The red arrow may indicate the loss of one allele of CDKN2A/B; Figure S4: GEO2R analysis of public dataset GSE19577, comparing three KMT2A::SEPTIN6-positive AML patients with 39 AML patients containing other types of KMT2A-rearrangement. A volcano-plot and the number of calculated statistically significant differentially expressed genes (N = 52) are shown above. Bar plots of selected genes including SEPTIN6, EMX2, MKX, BMPR1 B and HOXA7 are shown below; Table S1: List of 52 differentially expressed genesets obtained by GEO2R analysis of public dataset GSE19577, including downregulated SEPTIN6 and upregulated EMX2, BMPR1B and MKX.

## Abbreviations

The following abbreviations are used in this manuscript:

AML	Acute myeloid leukemia
BMP	Bone morphogenic protein
FISH	Fluorescence in situ hybridization
MLL	Mixed lineage leukemia
PTD	Partial tandem duplication
