# Supplementary material for: Fusion Gene KMT2A::SEPTIN6 in Acute Myeloid Leukemia Cell Line KOPM-88
Source: Cells. 2026 Jul 17;15(14):1286. doi: 10.3390/cells15141286 (PMC13406658; doi:10.3390/cells15141286)
Supplement: Supplementary file 1 [file cells-15-01286-s001.zip › cells-4388312-supplementary.pdf]

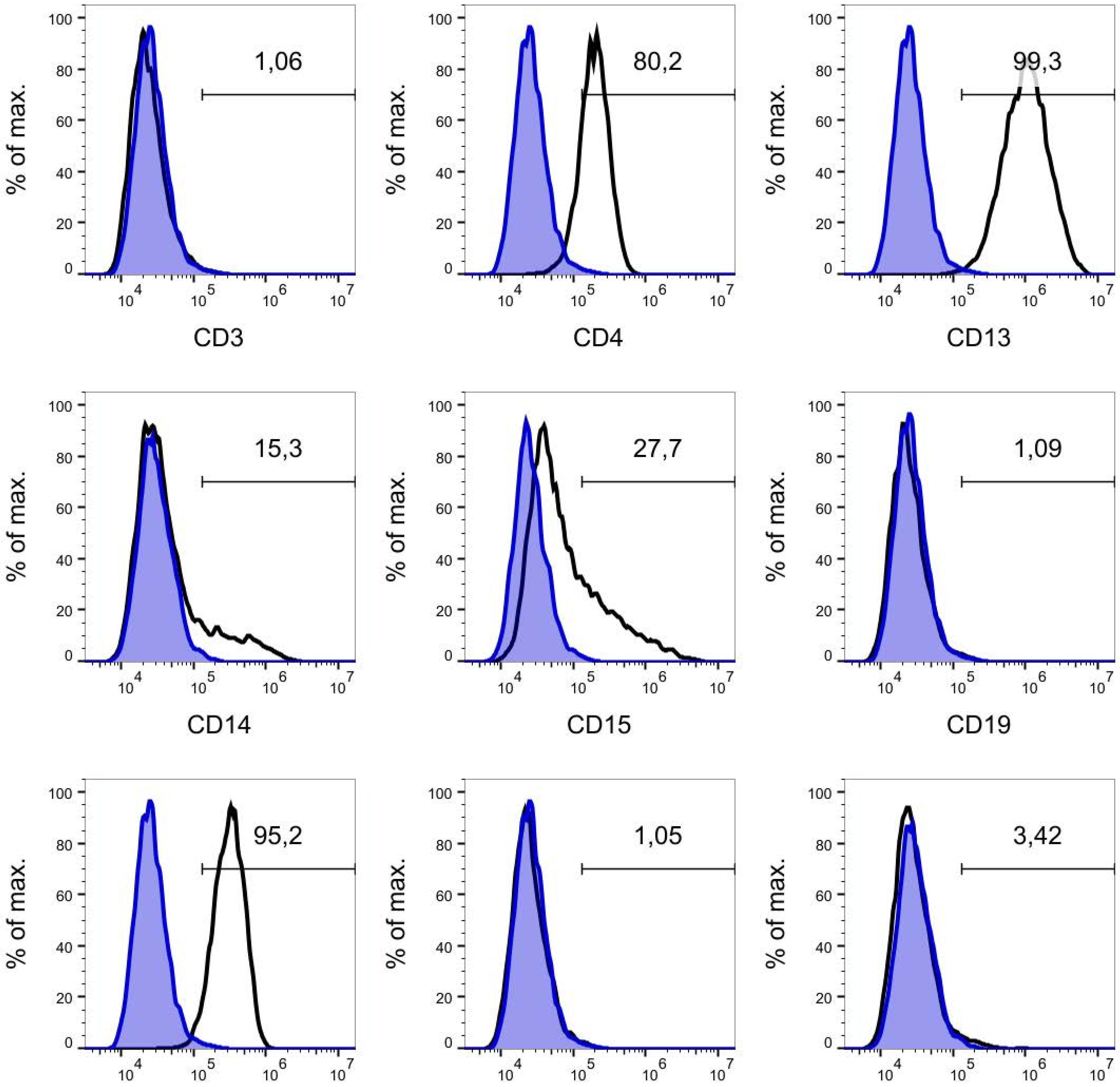

Figure S1: Flow cytometry analysis of KOPM-88 for CD3, CD4, CD13, CD14, CD15, CD19, CD33, CD34 and HLA-DR.

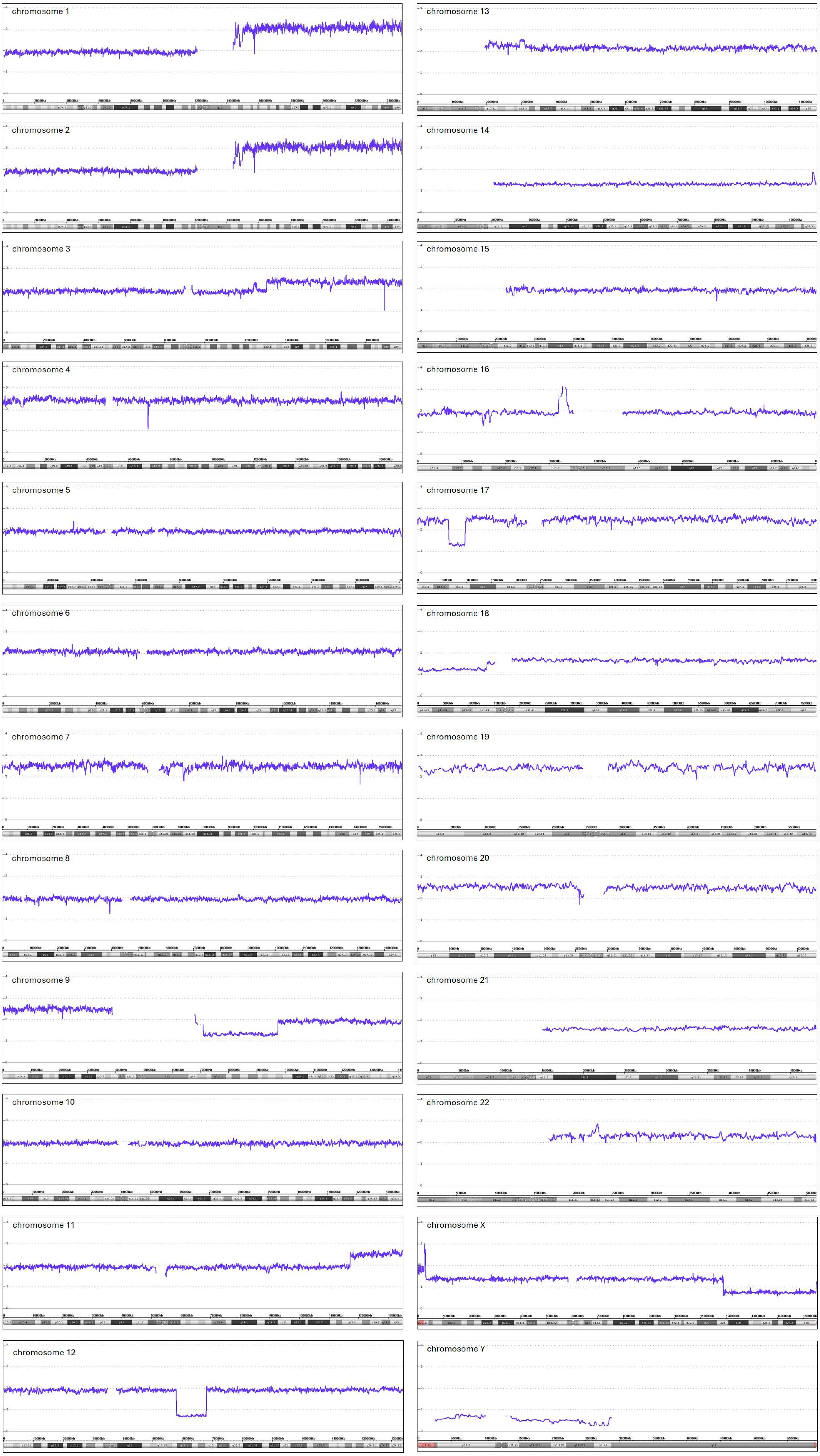

Figure S2: Genomic profiling data for KOPM-88, showing the copy number states of all chromosomes.

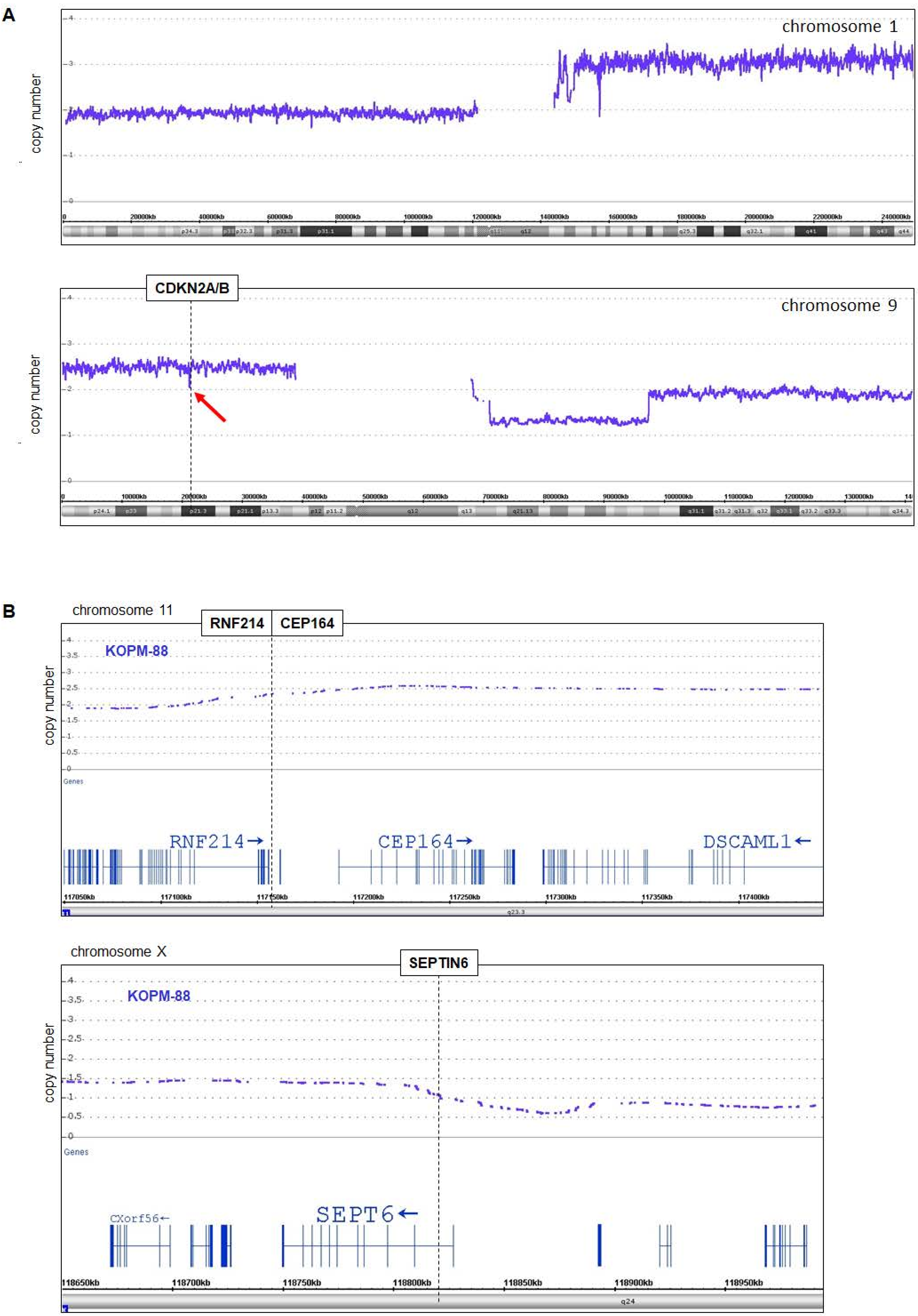

Figure S3: Genomic profiling data for KOPM-88, showing the copy number states of (A) chromosome 1 (above) and chromosome 9 (below), and (B) of enlarged parts of chromosome 11 (above) and chromosome X (below). The red arrow may indicate the loss of one allele of CDKN2A/B.

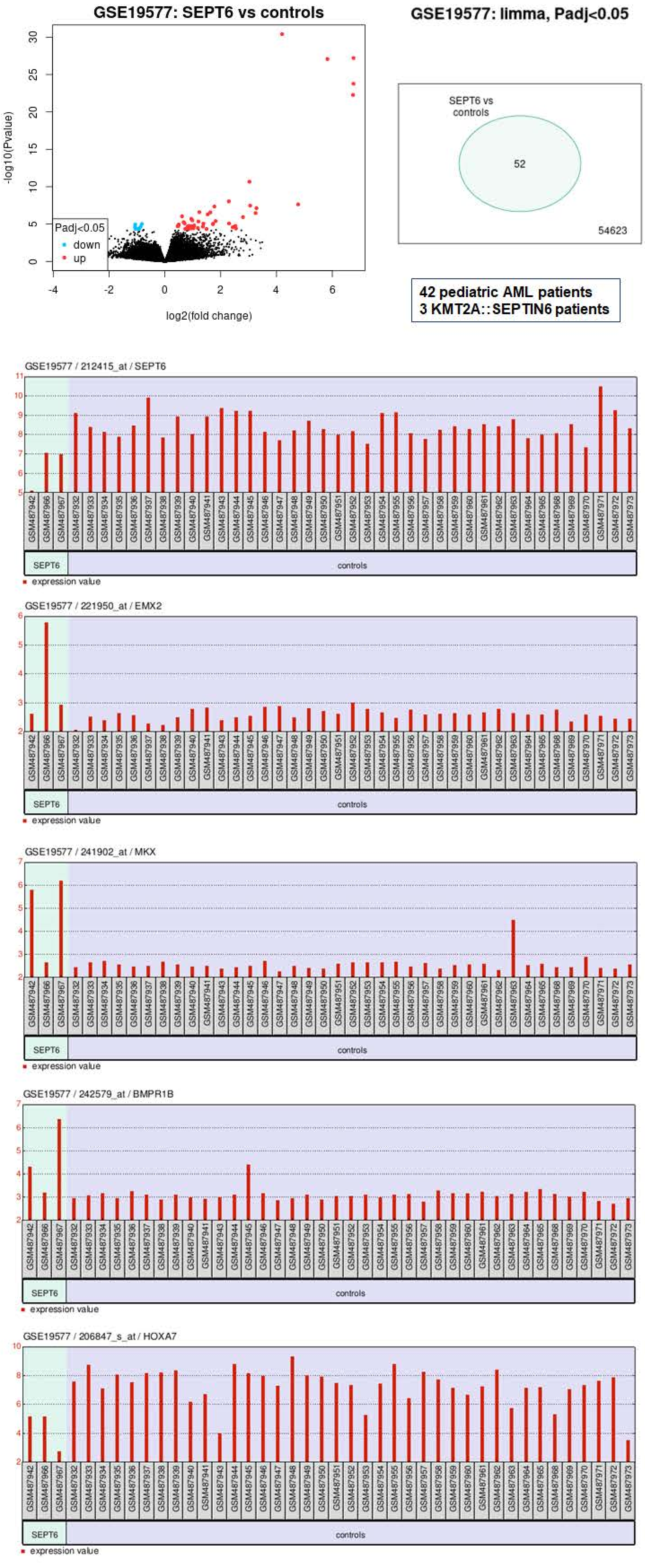

Figure S4: GEO2R analysis of public dataset GSE19577, comparing three KMT2A::SEPTIN6-positive AML patients with 39 AML patients containing other types of KMT2A-rearrangement. A volcano-plot and the number of calculated statistically significant differentially expressed genes (N=52) are shown above. Bar plots of selected genes including SEPTIN6, EMX2, MKX, BMPR1B and HOXA7 are shown below.

ID	Gene.symbol	Gene.title	log2(fold change)(SEPT6 vs controls)	-log10(Pvalue)(SEPT6 vs controls)
1553295_at	ABCA13	ATP binding cassette subfamily A member 13	0.73	4.358
1553994_at	NT5E	5'-nucleotidase ecto	0.962	4.439
1556147_at	LOC105377832	uncharacterized LOC105377832	0.852	4.753
1556194_a_at	HSPA4L	heat shock protein family A member 4 like	3.056	7.469
1556210_at			0.849	4.345
1557443_s_at	HNCAT21	head and neck cancer-associated transcript 21	0.494	4.943
1557845_at	DISC1FP1	DISC1 fusion partner 1 (non-protein coding)	1.363	4.659
1557891_s_at	STXBP5-AS1	STXBP5 antisense RNA 1	1.025	4.457
1558444_at			1.723	5.027
1559404_a_at	HMGN3-AS1	HMGN3 antisense RNA 1	0.671	5.253
1561886_a_at			0.614	6.036
1567681_at	SNORA74A	small nucleolar RNA, H/ACA box 74A	1.773	7.343
1567682_x_at	SNORA74A	small nucleolar RNA, H/ACA box 74A	1.38	5.062
1569608_x_at	LOC105379426	ankyrin repeat domain-containing protein 20A2	2.547	4.414
205543_at	HSPA4L	heat shock protein family A member 4 like	3.031	10.661
208358_s_at	UGT8	UDP glycosyltransferase 8	2.431	4.57
208675_s_at	DDOST	dolichyl-diphosphooligosaccharide	-0.946	4.345
208729_x_at	HLA-B	major histocompatibility complex, class I, B	-1.063	4.944
209140_x_at	HLA-B	major histocompatibility complex, class I, B	-0.825	5.004
210026_s_at	CARD10	caspase recruitment domain family member 10	0.948	5.696
211529_x_at	HLA-G	major histocompatibility complex, class I, G	-1.045	4.372

211911_x_at	HLA-B	major histocompatibility complex, class I, B	-1.072	4.595
212415_at	SEPT6	septin 6	-2.13	5.295
213894_at	THSD7A	thrombospondin type 1 domain containing 7A	6.76	23.769
214459_x_at	HLA-C	major histocompatibility complex, class I, C	-0.873	4.643
214920_at	THSD7A	thrombospondin type 1 domain containing 7A	6.762	27.198
215441_at			0.822	4.689
220325_at	TAF7L	TATA-box binding protein assoc. factor 7 like	1.494	4.333
220389_at	CCDC81	coiled-coil domain containing 81	1.202	5.348
220499_at	FNDC8	fibronectin type III domain containing 8	0.46	4.718
221950_at	EMX2	empty spiracles homeobox 2	1.182	4.467
222625_s_at	NDE1	nudE neurodevelopment protein 1	-0.926	4.356
224048_at	USP44	ubiquitin specific peptidase 44	2.524	4.751
225930_at	NKIRAS1	NFKB inhibitor interacting Ras like 1	1.236	6.587
226822_at	STOX2	storkhead box 2	2.801	5.932
229159_at	THSD7A	thrombospondin type 1 domain containing 7A	4.198	30.413
229975_at	BMPR1B	bone morphogenetic protein receptor type 1B	2.302	5.088
230008_at	THSD7A	thrombospondin type 1 domain containing 7A	5.827	27.07
230718_at	HSF5	heat shock transcription factor 5	1.821	5.404
231969_at	STOX2	storkhead box 2	4.777	7.632
232531_at	EMX2OS	EMX2 opposite strand/antisense RNA	1.049	4.804
233087_at	FBXL17	F-box and leucine rich repeat protein 17	1.725	5.002
234087_at			0.481	4.726

234317_s_at	STOX2	storkhead box 2	3.281	7.127
236221_at	AP4B1	adaptor related protein complex 4 beta 1	0.984	5.502
237137_at	SCARNA2	small Cajal body-specific RNA 2	1.63	6.569
238344_at			0.706	5.08
239468_at	MKX	mohawk homeobox	3.251	6.475
240818_at	OVCH1-AS1	OVCH1 antisense RNA 1	0.948	4.73
241902_at	MKX	mohawk homeobox	2.292	8.044
242579_at	BMPR1B	bone morphogenetic protein receptor type 1B	1.535	6.296
243756_at			6.744	22.271

Table S1: List of 52 differentially expressed genesets obtained by GEO2R analysis of public dataset GSE19577, including downregulated SEPTIN6 and upregulated EMX2, BMPR1B and MKX.
